# Pasta Structure Affects Mastication, Bolus Properties, and Postprandial Glucose and Insulin Metabolism in Healthy Adults

**DOI:** 10.1093/jn/nxab361

**Published:** 2021-10-20

**Authors:** Saara Vanhatalo, Margherita Dall'Asta, Marta Cossu, Laura Chiavaroli, Veronica Francinelli, Giuseppe Di Pede, Rossella Dodi, Johanna Närväinen, Monica Antonini, Matteo Goldoni, Ulla Holopainen-Mantila, Alessandra Dei Cas, Riccardo Bonadonna, Furio Brighenti, Kaisa Poutanen, Francesca Scazzina

**Affiliations:** VTT Technical Research Centre of Finland Ltd, Espoo, Finland; Department of Food and Drug, University of Parma, Parma, Italy; Department of Animal Science, Food and Nutrition, Università Cattolica del Sacro Cuore, Piacenza, Italy; Department of Food and Drug, University of Parma, Parma, Italy; Department of Nutritional Sciences, Temerty Faculty of Medicine, University of Toronto, Toronto, Ontario, Canada; Toronto 3D Knowledge Synthesis and Clinical Trials Unit, Clinical Nutrition and Risk Factor Modification Centre, St Michael's Hospital, Toronto, Ontario, Canada; Department of Food and Drug, University of Parma, Parma, Italy; Department of Food and Drug, University of Parma, Parma, Italy; Department of Food and Drug, University of Parma, Parma, Italy; VTT Technical Research Centre of Finland Ltd, Espoo, Finland; Department of Medicine and Surgery, University of Parma, Parma, Italy; Department of Medicine and Surgery, University of Parma, Parma, Italy; VTT Technical Research Centre of Finland Ltd, Espoo, Finland; Department of Medicine and Surgery, University of Parma, Parma, Italy; Department of Medicine and Surgery, University of Parma, Parma, Italy; Department of Food and Drug, University of Parma, Parma, Italy; VTT Technical Research Centre of Finland Ltd, Espoo, Finland; Department of Food and Drug, University of Parma, Parma, Italy

**Keywords:** structure, mastication, glycemic response, insulin response, C-peptide response

## Abstract

**Background:**

Structure and protein–starch interactions in pasta products can be responsible for lower postprandial glycemic responses compared with other cereal foods.

**Objectives:**

We tested the effect on postprandial glucose metabolism induced by 2 pasta products, couscous, and bread, through their structural changes during mastication and simulated gastric digestion.

**Methods:**

Two randomized controlled trials (*n* = 30/trial) in healthy, normal-weight adults (mean BMI of 23.9 kg/m^2^ (study 1) and 23.0 kg/m^2^ (study 2)) evaluated postprandial glucose metabolism modulation to portions of durum wheat semolina spaghetti, penne, couscous, and bread each containing 50 g available carbohydrate. A mastication trial involving 26 normal-weight adults was conducted to investigate mastication processes and changes in particle size distribution and microstructure (light microscopy) of boluses after mastication and in vitro gastric digestion.

**Results:**

Both pasta products resulted in lower areas under the 2-h curve for blood glucose (−40% for spaghetti and −22% for penne compared with couscous; −41% for spaghetti and −30% for penne compared with bread), compared with the other grain products (*P* < 0.05). Pasta products required more chews (spaghetti: 34 ± 18; penne: 38 ± 20; bread: 27 ± 13; couscous: 24 ± 17) and longer oral processing (spaghetti: 21 ± 13 s; penne: 23 ± 14 s; bread: 18 ± 9 s; couscous: 14 ± 10 s) compared with bread or couscous (*P* < 0.01). Pastas contained more large particles (46–67% of total particle area) compared with bread (0–30%) and couscous (1%) after mastication and in vitro gastric digestion. After in vitro gastric digestion, pasta samples still contained large areas of nonhydrolyzed starch embedded within the protein network; the protein in bread and couscous was almost entirely digested, and the starch was hydrolyzed.

**Conclusions:**

Preservation of the pasta structure during mastication and gastric digestion explains slower starch hydrolysis and, consequently, lower postprandial glycemia compared with bread or couscous prepared from the same durum wheat semolina flour in healthy adults.

The postprandial in vivo trials were registered at clinicaltrials.gov as NCT03098017 and NCT03104686.

See corresponding editorial on page 920.

## Introduction

Starch digestibility and glycemic response to carbohydrate-rich foods can vary according to the structural properties (molecular, microstructure, macrostructure) of food (1). The food matrix modulates both structure disintegration and enzymatic hydrolysis of starch in the gastrointestinal tract (2). Therefore, products with similar ingredients, but different processing methods and structures, can induce very different postprandial responses. Salivary α-amylase initiates starch digestion in the mouth during chewing (3). Activity of salivary α-amylase has been suggested to continue in the stomach, until the low pH inactivates the enzyme. The last step of starch digestion takes place in the small intestine by pancreatic α-amylase and brush border enzymes.

Pasta products, couscous, and bread are all sources of starch in the diet that can be prepared using semolina flour as the main ingredient. The manufacturing process of each product starts with wetting and mixing of semolina flour. In wheat bread baking, the dough is leavened with yeast and baked. During mixing, gluten forms a network (4) retaining gas bubbles in the dough, thus creating a porous structure. Couscous is manufactured by mixing semolina with water to form agglomerates followed with sifting, steam-cooking, and drying (5), and it is prepared by adding boiled water and letting it stand for ∼5 min. Its granules are dense in structure, but due to the small granule size, couscous has a high surface-to-weight ratio. The pasta-making process involves 3 steps, namely mixing, forming (by extrusion), and drying (6). During the forming step, the crumbly dough, in which gluten is barely formed, is pushed by a screw to the head press on which a die is set. The dough is constantly submitted to shearing stress that promotes the formation of the protein network but, most of all, as the dough is transferred, the extrusion pressure builds up and this is essential to give the product a highly compact structure (6). Through extrusion, pasta reaches its final shape, with a low surface-to-weight ratio. During the drying step, cross-linking between the different gluten proteins is promoted, thus leading to more intense starch encapsulation by the gluten network (6). As a consequence of this technological process, the microstructure of pasta is compact and relatively dense (7, 8). Finally, cooking in excess water gives pasta its ultimate structure, which is described as a compact matrix of gelatinized starch strongly entrapped in a protein (gluten) network.

In vitro studies have shown that pasta contains relatively little rapidly digestible starch and the starch is mostly slowly digestible, different from other starchy foods such as bread (9). The slow rate of starch digestion of pasta is reflected in its relatively low glycemic index (GI) (10–12). Slow starch digestibility has been suggested to result from the structural properties of pasta at macroscopic (low surface-to-weight ratio), microscopic (encapsulation of starch by proteins), and molecular (retrogradation of starch, complex formation) levels (6).

The aim of this study was to explain the postprandial glycemia features of 2 pasta products, couscous, and bread by their structural changes during mastication and gastric digestion. Differences between the 2 pasta shapes, spaghetti and penne, were also investigated. The selection of 2 different shapes of dried pasta was based on addressing potential differences resulting from pasta shapes and sizes on structure disintegration, starch digestibility, and postprandial glucose metabolism; moreover, commercial data from the sponsor of the study indicated that these are the most highly consumed types of pasta on the market.

## Methods

### Study products

**FIGURE 1:**
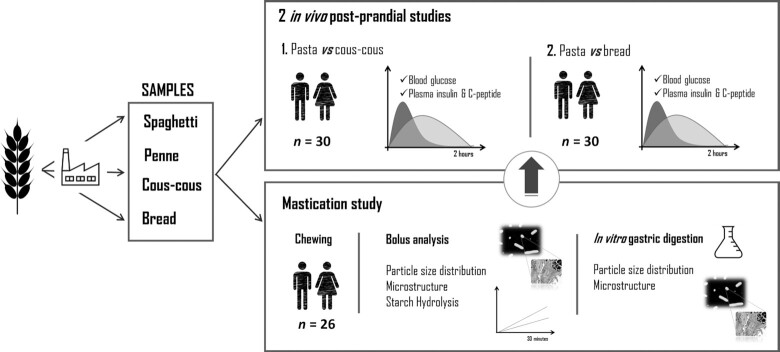
Design of the study.

Four durum wheat semolina products with the same ingredients but different structures were studied in 2 postprandial trials and 1 mastication trial (**Figure 1**). The products were: long pasta (spaghetti), short pasta (penne), couscous, and bread. Pasta products (short pasta PENNE and long pasta SPAGHETTI n.5), COUS COUS, and SEMOLINO, which was used for bread preparation, were industrially manufactured by Barilla G. e R. F.lli S.p.A. and are commercially available in Europe. Pastas and couscous were cooked individually for each participant just before each study visit. A single portion of spaghetti or penne was boiled in 1.5 L boiling water with the addition of 7 g salt for 9 and 11 min, respectively, according to the package instructions. The couscous dish was prepared by pouring boiling water on the couscous (1:1 v/v), mixing gently, and letting it stand for 5 min. Bread was baked in a bread machine (La Panetteria 151936; Princess Silver) following the recipe: 500 g semolina, 300 g water, 10 g salt, 7 g dried yeast (Mastrofornaio, PANEANGELI). For each visit day, the bread was prepared fresh and left to cool at room temperature. In the mastication trial the bread was baked 1–4 h before study visits and cut in cubes including crust on 1 side. In the postprandial trial, the bread was left to cool at room temperature for ∼8 h, and each portion of bread was sliced excluding the sides of the loaf to ensure an equal crust/inner bread ratio. The nutritional composition of the products is detailed in **Table 1**.

**TABLE 1:** Nutritional composition of the food products

	Spaghetti	Penne	Bread	Couscous	Glucose monohydrate
Energy, kJ/100 g	1506	1515	1008	1498	1521
Protein, g/100 g	13.6	13.9	9.4	12.9	—
Carbohydrate, g/100 g	70.7	70.1	46.0	71.0	90.9
Sugar, g/100 g	2.5	2.6	2.2	1.7	90.9
Fat, g/100 g	1.8	1.9	1.2	1.8	
Saturated fat, g/100 g	0.4	0.4	0.3	0.4	—
Fiber, g/100 g	3.5	3.9	3.9	3.0	
Sodium, g/100 g	0.004	0.002	0.546	0.003	—

### Postprandial in vivo studies

Two postprandial in vivo trials were conducted at the Department of Food and Drug and Department of Medicine and Surgery of Parma University/Hospital of Parma (Azienda Ospedaliero-Universitaria di Parma), in accordance with the Declaration of Helsinki, Good Clinical Practice (CPMP/ICH/135/95) and the European regulatory requirements (Directive 75/78/CE). The study protocols were both approved by the local ethics committee (Comitato Etico per Parma: protocol numbers 6659 and 6654), and written informed consent was obtained from each participant for both trials, prior to starting the study. The trials were registered on www.clinicaltrials.gov (registration numbers: NCT03098017 and NCT03104686). Both were randomized, controlled, single-blinded, crossover, acute postprandial clinical trials conducted based on the ILSI (International Life Sciences Institute)guidelines for nutritional studies (13) and followed the ISO 26642:2010 guidelines for the GI determination of food products (14).

In the first trial (study 1), long pasta (spaghetti), short pasta (penne), couscous, and a glucose solution (control) were administered to 30 participants. In the second trial (study 2), long pasta (spaghetti), short pasta (penne), bread, and a glucose solution (control) were administered to 30 participants.

#### Participants

Female and male participants for conducting both studies were recruited in Parma (Italy). Randomly selected participants met prespecified criteria before enrollment into each postprandial trial. The exclusion criteria were set based on the ISO 26642:2010 guidelines (14) with some modifications: age <18 y, BMI >30 kg/m^2^, celiac disease, metabolic disorders (e.g., diabetes, hypertension, dyslipidemia, glucose intolerance), chronic use of medications for any conditions (including psychiatric diseases), use of dietary supplements affecting metabolism, and anemia. Once enrolled, participants were assessed for blood pressure, weight, BMI, and waist circumference. Body weight was checked at each visit to monitor weight gain or loss throughout the trial. Baseline characteristics of the participants are reported in **Supplemental Table 1**. The flow diagram of the enrollment is reported in **Supplemental Figure 1**. All the participants enrolled completed the study without any protocol violations. A randomization table was generated using a randomized block design and the order of the test meals was determined randomly according to a computerized random number generator (https://www.randomizer.org/). This number table was made by a person not involved in subject enrollment, and using a numbered sequence in sealed, opaque envelopes. The list was blinded for the principal investigator and participants. The participants were randomly assigned to the study on the day of their first visit.

#### Procedure

The 2 postprandial in vivo trials each consisted of 4 separate occasions during which participants attended clinical testing and randomly consumed test meals with a minimum washout of 1 wk. The day before each visit, participants were instructed to avoid foods that might interfere with glucose metabolism the next day, based on the indication of the ISO 26642:2010 (14), and others rich in indigestible carbohydrates to avoid the second meal effect (15). Upon arrival at the hospital (08:30 to 09:00) where clinical testing took place, a cannula was inserted in the vein of the forearm by the medical doctor and 2 fasting blood samples were taken 10 min apart. Venous blood was collected for the determination of insulin and C-peptide while, in parallel, 250 μL capillary blood were collected by finger prick (ACCU-CHEK Safe-T-Pro Plus; Roche) in a precoded polypropylene tube with EDTA and glyceraldehyde [10 μL mixed water stock solutions of glyceraldehyde (3%w/v) and EDTA (10% w/v)] for blood glucose analysis. Immediately after meal preparation, participants were instructed to consume the test meal together with 500 mL still bottled water over 12 min.

Test meals were portioned to contain 50 g available carbohydrates (penne and spaghetti 71 g, 70 g couscous, 109 g bread) and no addition of condiments was allowed; for the control meal a glucose solution was freshly prepared dissolving 55 g glucose monohydrate (Farmalabor) with 250 mL water. All participants were seated in the same room and asked to remain seated throughout testing, and venous and capillary blood were collected at specific postprandial timepoints (15, 30, 45, 60, 90, and 120 min) after the meal consumption. Venous samples were immediately processed, and plasma for insulin and C-peptide determination was obtained after centrifugation at 2000 × *g* for 10 min at 4°C, transferred into polypropylene tubes, and stored at −70°C until the analysis, performed by means of specific immunoassays kits (ELISA) (Insulin REF: 10-113-10; C-peptide REF: 10-1136-01; Mercodia), following the manufacturer's instruction. Glucose determination was done using a YSI model 2300 analyzer (Yellow Springs Instrument Company). Blinding of the participants or the personnel involved in the trial delivering the test meals was not possible due to the nature and physical form of the foods tested in the trial. To reduce potential bias, personnel involved in randomization, in the analyses of the samples collected, and in the data processing were blinded.

### Mastication study

#### Participants

Females and males (*n* = 26) aged 18–75 y and BMI <30 were recruited in the Otaniemi (Espoo, Finland) campus area. Volunteers with (self-reported) anemia, glucose intolerance or diabetes, hypertension, dyslipidemia, or celiac disease were excluded as well as those undergoing very intense physical activity. Gender distribution, age, and BMI of the participants are presented in **Supplemental Table 2**.

The Coordinating Research Ethics Committee of the Helsinki and Uusimaa Hospital District approved the study protocol. The study followed the ethical principles of good research and clinical practice described in the Declaration of Helsinki.

#### Procedure

The participants were instructed to eat ad libitum breakfast 1–1.5 h before the study visit. The breakfasts of the participants were not uniform. The experiment followed a crossover, single-blind design. On the study visit, all 4 food samples were served, in a random order, and each food type was served in 3 portions. Portion sizes were determined to represent a mouthful of food. One portion size was 8 g each for spaghetti and penne (2.6 g and 2.4 g carbohydrates), ∼9 g as 2 × 2 × 2-cm cubes of bread (4 g carbohydrates), and 7 g (2.2 g carbohydrates) for couscous. The participant was asked to masticate each portion until he/she considered it ready for swallowing and to expectorate it to a plastic container, which was kept on ice. There was a 2-min break between each food type. Finally, 3 pieces of chewing gum were served in a row and the participant was asked to chew each piece for 20 s. The data on oral processing of chewing gum were used as a reference for mastication force parameters. Bolus samples were stored at −70°C.

#### Mastication assessment with electromyography

The mastication process was characterized by measuring the electrical activity of facial muscles by electromyography (EMG) equipment (Mega Electronics) following the procedure described earlier by Pentikäinen et al. (16). The parameters derived from EMG data were: the duration of oral processing (seconds), the duration of EMG activity (seconds), the number of chews, relative chewing force (highest EMG amplitude for the product normalized to the highest EMG amplitude for chewing gum), and relative work (time of EMG activity × relative chewing force). Averaged values from the 4 muscles were used. All analyses were done using in-house build scrips in Matlab (The MathWorks Inc).

#### Bolus sample pooling and determination of saliva in food bolus samples

Average masticators of the 26 study participants were defined based on the mastication process of bread (the duration of oral processing and the number of chews) in the mastication trial. Bolus samples of 12 average masticators were used for further assessment. Bolus samples were gently melted and pooled to obtain 1 bolus sample for each food product. Fresh food samples and bolus samples (BSs) were dried in an oven at 105°C overnight resulting in dry samples (DSs). The moisture contents of the food samples and BSs were determined by BS − DS/BS × 100. The amount of saliva in the BS was determined as the difference of the moisture content between the bolus and food samples.

#### Disintegration of food matrix after mastication and after in vitro gastric digestion

Untreated pooled food BSs were diluted into 100 mL water and mixed with magnetic stirring for 25 min. Part of the BS was treated with pepsin (in vitro gastric digestion): Pooled food BSs with 0.8 g dry matter were mixed with 10 mL 0.05 M Na-K phosphate buffer (pH 1.5) with 0.25% pepsin (w/v) and pH was adjusted to 1.5. The mixture was incubated with magnetic stirring at 37°C for 30 min. Pooled BSs after in vitro gastric treatment were rinsed with 100 mL cold tap water.

The particles present in these 2 resulting solutions (untreated BSs and in vitro gastric-digested samples) were allowed to settle for 5 min. The turbid liquid containing mostly the particles with area <0.03 mm^2^ was removed. The sample volume was adjusted with water up to 100 mL. The liquids containing the particles were poured on petri dishes and adjusted so that contact among particles was minimized. Digital images were taken of each petri dish (6 to 10 petri dishes per sample). Images were calibrated and particle areas were detected using Cell^P imaging software (Olympus). The area occupied by different size particles of the total area was determined.

#### Microstructure of food products after mastication and after in vitro gastric digestion

For microscopy, aliquots of the pooled boluses were separated. In vitro gastric-digested samples for microscopy were collected from the particle separation step after incubation with pepsin as described in the previous paragraph. To enable their handling, the samples were first embedded in 2% (w/v) agar and then fixed in 1% (v/v) glutaraldehyde in 0.1 M Na-K phosphate buffer (pH 7.0), dehydrated in a graded ethanol series, and embedded in hydroxyethyl methylacrylate resin as recommended by the manufacturer (Leica Historesin embedding kit; Leica Microsystems). Polymerized samples were sectioned (2-μm sections) in a rotary microtome HM 355S (Microm Laborgeräte GmbH) using a tungsten carbide knife. The sections were transferred onto glass slides and stained. Protein was stained with aqueous 0.1% (w/v) Light Green SF (BDH Chemicals Ltd) for 1 min, and starch with 1:10 diluted Lugol iodine solution (I_2_ 0.33% w/v and KI 0.67% w/v). When imaged in brightfield, protein stained with Light Green appears green. Iodine stains the amylose component of starch blue and amylopectin brown. Therefore, ungelatinized starch is detected as dark purple whereas gelatinized starch is typically light blue or purple. The stained sections were examined with a Zeiss Axio Imager M.2 microscope (Carl Zeiss GmbH). Micrographs were obtained using a Zeiss Axiocam 506 CCD color camera (Zeiss) and the Zen imaging software (Zeiss). Representative images were selected for publication.

#### Mastication-induced post vivo starch hydrolysis

The rate of mastication-induced starch hydrolysis was determined with a method modified from Granfeldt et al. (17) and described earlier by Pentikäinen et al. (18). BSs containing 0.5 g starch were transferred to dialysis tubing (Spectra/Por No. 2, flat width 45 mm, molecular weight cutoff 12−14 kDa) with 15 mL cold phosphate buffer (pH 6.9). The tubing was first incubated in a beaker with 0.05 M phosphate buffer (400 mL) at 37°C for 30 min, with magnetic stirring. The salivary α-amylase present in the BSs initiated starch hydrolysis. Aliquots each of 2 mL were removed at time points 0, 1.5, 3, 6, 9, 12, 15, and 30 min, and frozen (−20°C). The removed samples were further incubated with amyloglucosidase (Megazyme)) at 50°C for 15 min to hydrolyze the solubilized starch to glucose. Free glucose was determined by treating the samples with glucose oxidase peroxidase reagent (Megazyme) for 20 min, and by reading the absorbance at 510 nm. Glucose solution (100 μg/0.1 mL) was used as a standard. The amount of released glucose was converted to starch by multiplying with 0.9. The degree of starch hydrolysis was calculated as the proportion of the released starch from the original starch content of the BS. The rate of starch hydrolysis (percentage per minute) for each BS was calculated as (proportion of released starch at 30 min − proportion of released starch at 0 min)/30.

### Statistical analyses

#### Postprandial in vivo trials

The primary outcomes in both trials were the difference in the incremental AUC_0–120_ for glucose and insulin after consumption of the pasta products compared with couscous (in vivo study 1) or bread (in vivo study 2), whereas the secondary outcome was the difference in the incremental AUC_0–120_ for C-peptide. The primary statistical analysis was the between-treatments difference in AUC_0–120_ for postprandial glucose and insulin between food products including all randomized participants (intention-to-treat analysis). The sample size was assessed based on data for the AUC_0–120_ for glucose obtained from a previous publication (10), and data for the AUC_0–120_ of insulin obtained from unpublished work from our group (study approved by the ethics committee—Comitato Etico per Parma; number of protocol: 22669; clinicaltrial.gov identifier: NCT03024983). To preserve the family-wise type I error rate, the adjustment for multiple outcomes was considered in the sample size calculation. Thirty subjects were required to ensure a power >90% with α = 0.025 for glucose, whereas 12 subjects were required to achieve 90% power to detect a mean of paired differences with α = 0.025 for insulin. In each case, the incremental AUC_0–120_ for blood glucose, insulin, and C-peptide was geometrically calculated using the trapezoidal method, ignoring the area beneath the baseline level as suggested by ISO 26642:2010 (14). Incremental postprandial curves were obtained by calculating the increment for each timepoint by comparing each absolute value with the baseline concentration, which was calculated as the mean concentration of glucose measured in the samples taken during fasting at times −10 and 0 min. Data are presented as mean ± SEM, unless otherwise stated. All data collected were assessed for normality using the Kolmogorov–Smirnov test. Postprandial changes in plasma glucose, insulin, and C-peptide expressed as AUC_0–120_ and incremental postprandial curves (0–120 min) calculated for each food product were analyzed using the general linear model for repeated measures. Greenhouse–Geisser correction for degrees of freedom was used when the Mauchly test of sphericity was significant. The Bonferroni post hoc test was used for multiple comparisons. Statistical significance was determined at *P* < 0.05. Statistical analyses were run with SPSS for Windows applications (Version 25.0; SPSS Inc).

#### Mastication trial

For mastication experiments, paired *t* tests were conducted to evaluate the between-product differences in each mastication parameter. A *P* value <0.05 was considered statistically significant.

## Results

### Postprandial in vivo trials

#### Glucose responses

**FIGURE 2:**
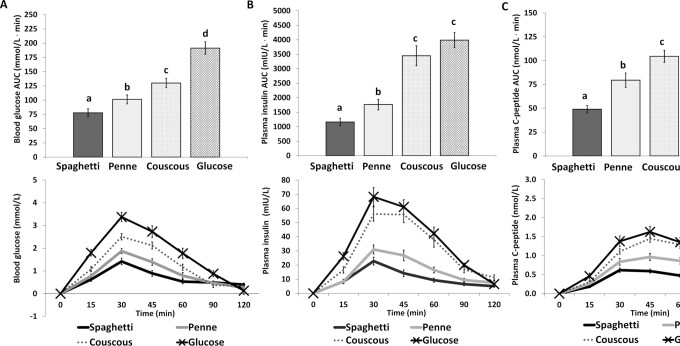
Incremental AUC and incremental postprandial curve (0–120 min) for capillary blood glucose (A), plasma insulin (B), and plasma C-peptide (C) for pasta products (penne and spaghetti), couscous, and glucose, all consumed at 50 g available carbohydrate (*n* = 30) (study 1). Data are expressed as mean ± SEM. Labeled means in the bars without a common letter differ, *P* < 0.05.

**FIGURE 3:**
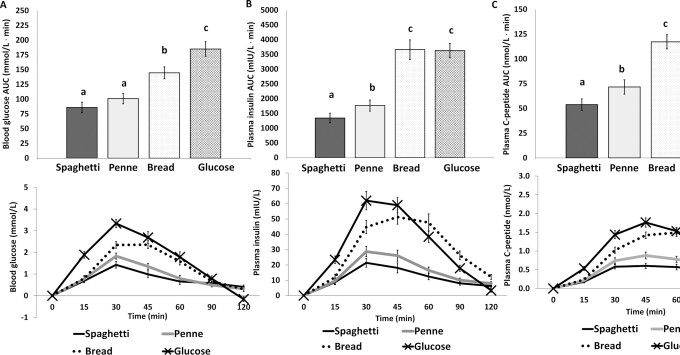
Incremental AUC and incremental postprandial response curve (0–120 min) for capillary blood glucose (A), plasma insulin (B), and plasma C-peptide (C) for pasta products (penne and spaghetti), bread, and glucose, all consumed at 50 g available carbohydrate (*n* = 30) (study 2). Data are expressed as mean ± SEM. Labeled means in the bars without a common letter differ, *P* < 0.05.

**Figures 2**A and **3**A present the postprandial glucose responses of the food products in both trials. Both pasta products (penne and spaghetti) induced a lower postprandial glucose response when compared with either couscous (*P* < 0.05 for penne compared with couscous; *P* < 0.001 for spaghetti compared with couscous) or bread (*P* < 0.005 for penne compared with bread; *P* < 0.001 for spaghetti compared with bread). Finally, spaghetti induced a lower postprandial glucose response (AUC_0–120_) than penne (*P* < 0.05) only in study 1. The AUC_0–120_ for glucose was significantly lower for each food product compared with the glucose solution control (*P* < 0.001 compared with penne, spaghetti, in both studies, and compared with couscous; *P* < 0.05 compared with bread) (Figures 2A and 3A).

#### Insulin responses

Figures 2B and 3B present the postprandial insulin responses of the food products in both trials. Both pasta products induced lower postprandial insulin responses compared with either couscous or bread (*P* < 0.001 for penne or spaghetti compared with couscous or bread). The AUC_0–120_ for insulin was lower for spaghetti compared with penne (*P* < 0.001 in study 1 and *P* < 0.05 in study 2). The AUC_0–120_ for insulin was significantly lower for penne and spaghetti than for the glucose solution (*P* < 0.001 for both comparisons in both studies), whereas postprandial insulin responses for couscous or bread did not differ from the glucose solution.

#### C-peptide responses

Figures 2C and 3C present the postprandial C-peptide responses of the food products in both trials. Both pasta products induced lower postprandial C-peptide responses compared with either couscous or bread (*P* < 0.05 for penne compared with couscous; *P* < 0.001 for spaghetti compared with couscous; *P* < 0.001 for penne or spaghetti compared with bread). Spaghetti induced a lower postprandial C-peptide response than penne (*P* < 0.001 in study 1; *P* < 0.005 in study 2). Postprandial C-peptide response was significantly lower for penne and spaghetti than for the glucose solution (*P* < 0.05 for penne compared with glucose solution; *P* < 0.001 for spaghetti compared with glucose solution in study 1, and *P* < 0.001 for penne or spaghetti compared with glucose solution in study 2). Postprandial C-peptide responses for couscous or bread did not differ from the glucose solution.

### Mastication process and bolus properties

#### Mastication process

**FIGURE 4:**
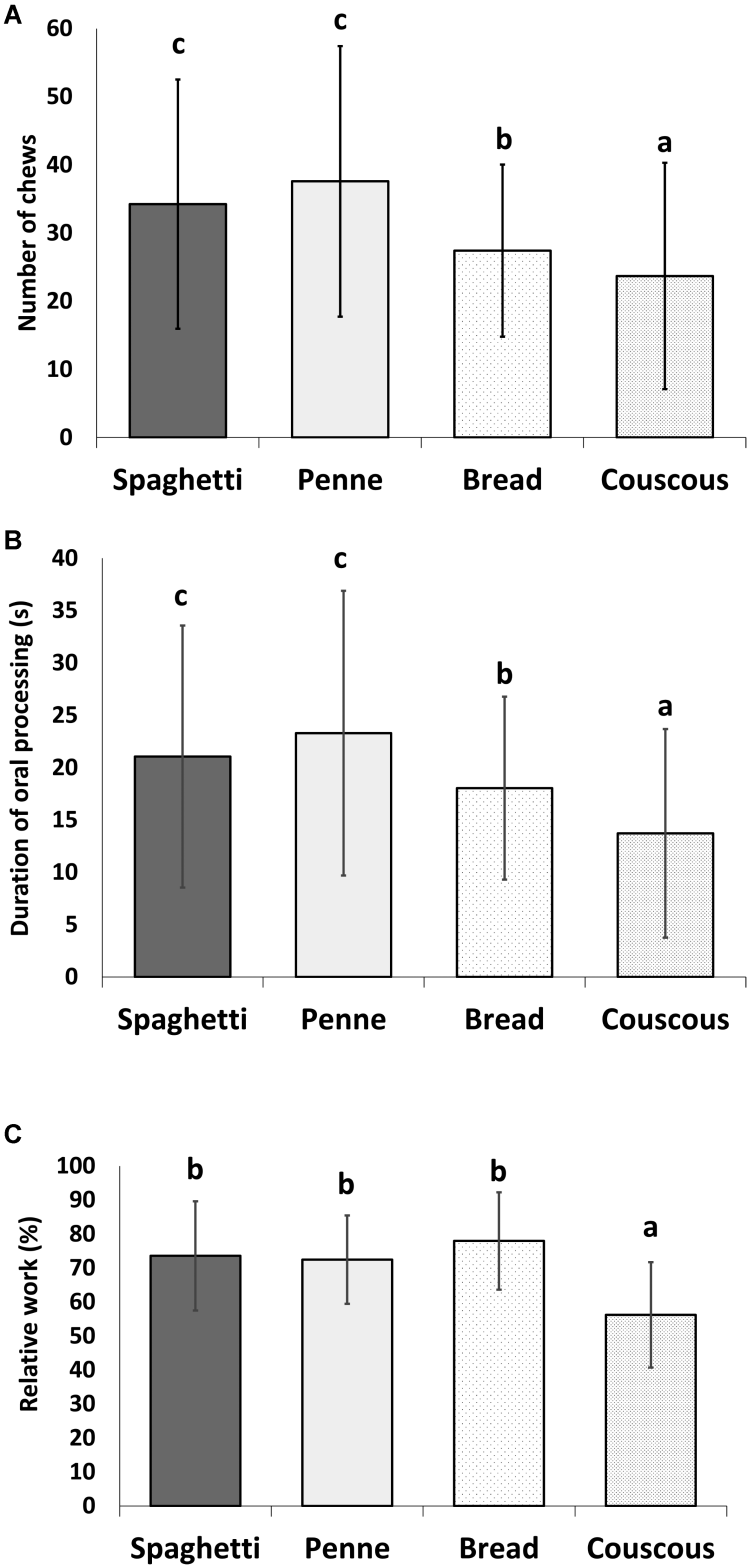
Average mastication parameters (*n* = 26) for mouthful of samples for (A) number of chews, (B) duration of oral processing (seconds), and (C) relative work (%, calculated relative to the work used for chewing gum). The error bars shown are average of SDs for the 4 muscles analyzed. Labeled means in the bars without a common letter differ, *P* < 0.05.

Both pasta products required more chews than bread or couscous, which was reflected in the duration of oral processing (*P* < 0.01 for all comparisons) (**Figure 4**). Bread was chewed slightly more than couscous (*P* < 0.05, for number of chews; *P* < 0.01, for duration of oral processing). The work needed to masticate was significantly lower for couscous compared with all other food products (*P* < 0.01). There were no differences between the 2 pasta products in mastication parameters.

#### Amount of saliva in boluses

The amount of saliva in pasta boluses was lower than the amount of saliva in either the bread bolus or couscous bolus (**Table 2**). The initial moisture contents of pastas and couscous were higher than that of bread.

**TABLE 2:** Moisture content of food and pooled bolus samples and amount of saliva in bolus samples

Food	Moisture content of food, %	Moisture content of pooled bolus, %	Amount of saliva in bolus, g/g
Spaghetti	58.2	62.5	4.4
Penne	52.9	59.9	7.0
Bread	33.7	55.9	22.2
Couscous	55.2	64.4	9.2
Range	33.7–58.2	55.9–64.4	4.4–22.2

#### Disintegration of food matrix after mastication

**FIGURE 5:**
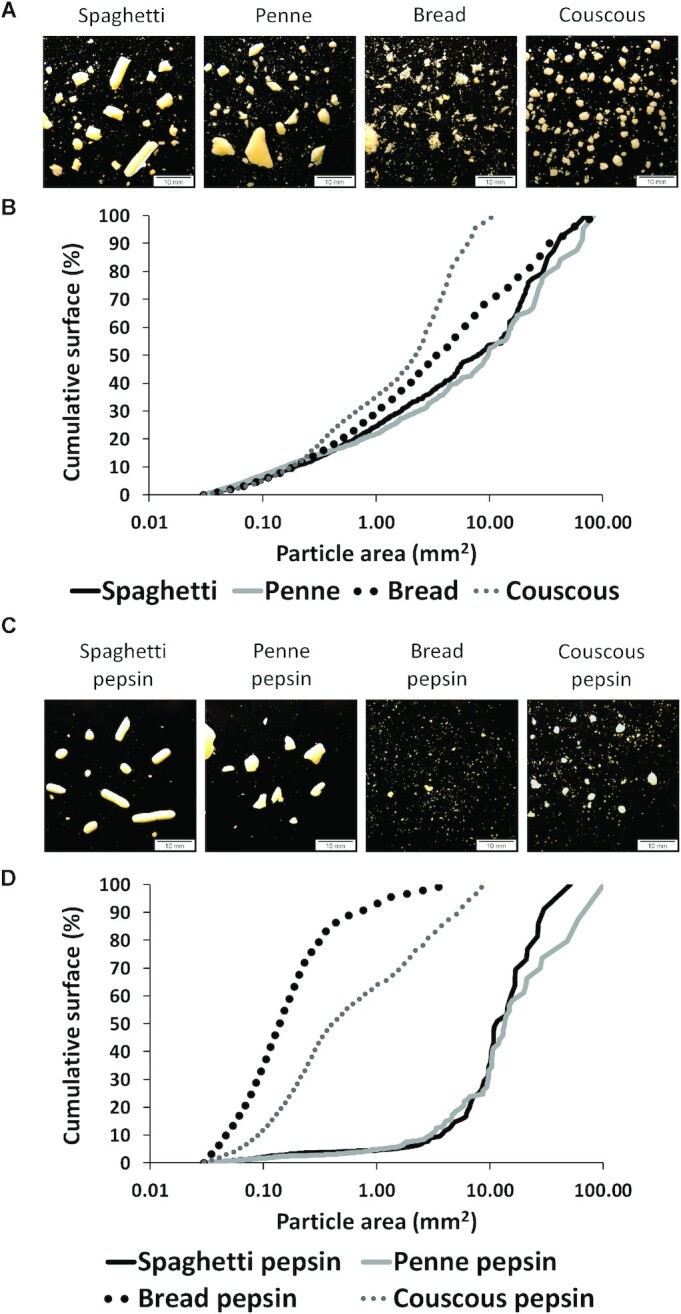
Photographs of pooled boluses (*n* = 12) after mastication (A) and after in vitro gastric digestion (C), and particle area distribution of the same samples (B and D). The curves represent a cumulative percentage of the total area occupied by particles. Values for particle area are logarithmic.

The BSs differed with respect to the proportions of medium size (1−10 mm^2^) and large (>10 mm^2^) particles of the total particle area, whereas no differences were seen in particles <0.1 mm^2^ (**Figure 5**). Pasta boluses contained the highest proportion of large particles whereas the couscous bolus contained the highest proportion of the smallest particles. The bread bolus had the highest proportion of medium-size particles (1−10 mm^2^). The amount of dry matter lost as the smallest particles (<0.02 mm^2^) in the turbid liquid after particle separation was highest in the bread bolus (8%). Pasta and couscous bolus particles were smooth-rimmed, whereas the appearance of the bread bolus particles was shredded.

#### Microstructure of boluses

**FIGURE 6:**
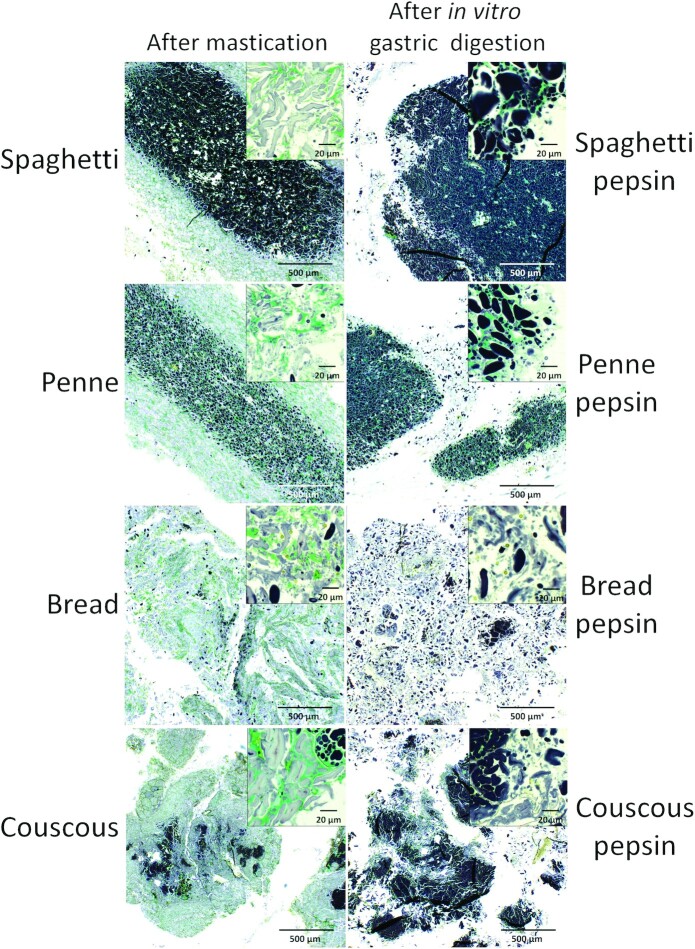
Light micrographs of pooled boluses (*n* = 12) after mastication and after in vitro gastric digestion. Protein appears green (stained with Light Green) and starch granules purple (stained with Lugol iodine). Scale bar is 500 μm in the main figures showing the overall appearance of the bolus particles, and 20 μm in the subfigures presenting the outermost layer of bolus particles.

All the boluses contained a protein network in which starch granules were located (**Figure 6**). Spaghetti and penne boluses contained large, distinct particles (<1 mm) with darkly stained, partially gelatinized starch granules present in the innermost part of the particle. The border between the innermost part with partially gelatinized starch and external region with swollen and deformed starch granules was clear. In the couscous bolus, starch granules were largely swollen and deformed. However, there were darkly stained starch granules present in the coarse flour particles with intact cellular structure. Starch granules in the bread bolus were thoroughly deformed and swollen. Darkly stained, little modified starch granules were detected also in the bread bolus, but they were fewer and more scattered compared with the pastas or couscous. The starch granules in the outer layers of the bolus particles (Figure 6, higher magnification) were swollen and deformed for the most part.

BSs differed from each other in their degree of compactness. In the spaghetti bolus, the least modified starch granules were the most tightly packed whereas in the penne bolus more protein and air spaces were observed between these starch granules.

#### Mastication-induced post vivo starch hydrolysis

**FIGURE 7:**
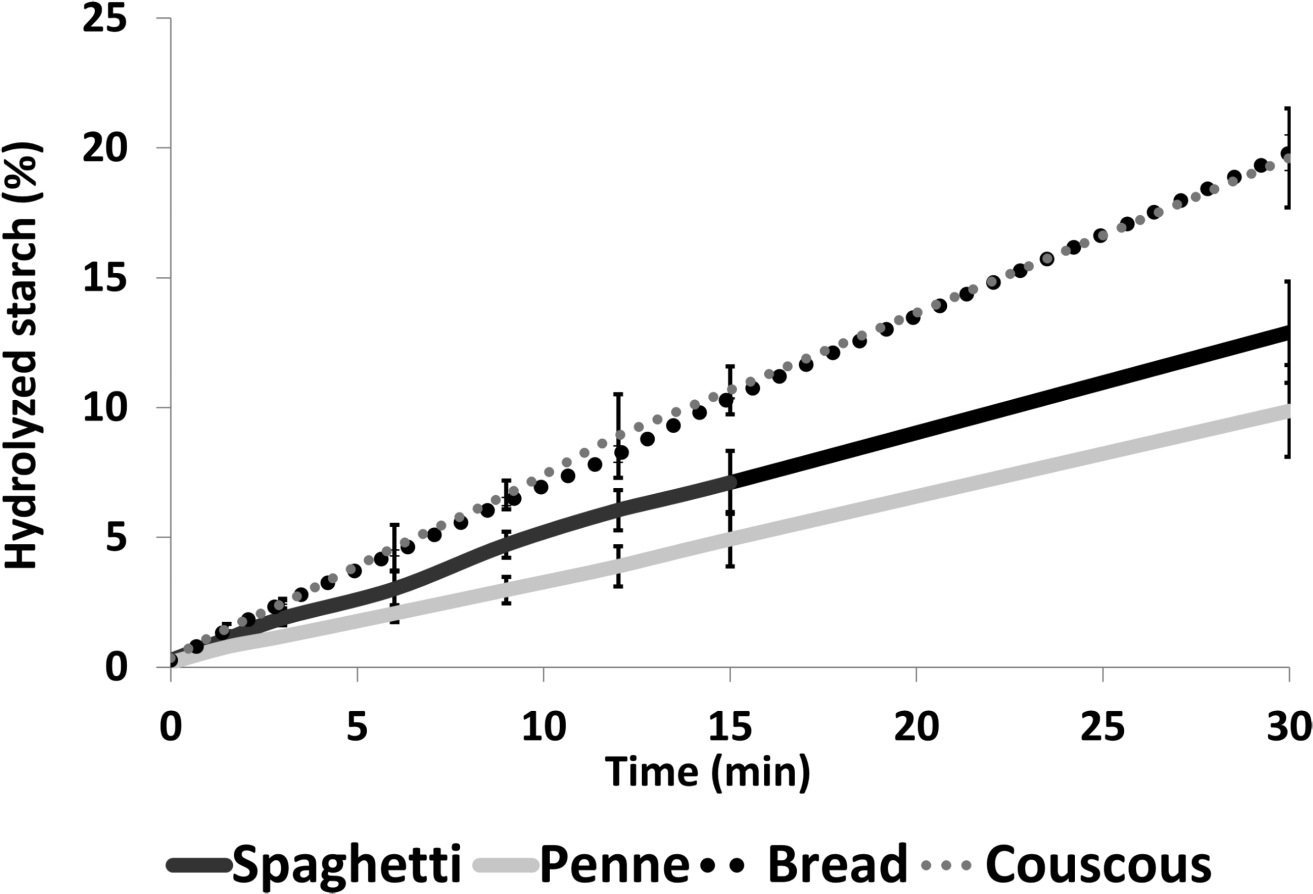
Mastication-induced post vivo starch hydrolysis during 30-min incubation. Values (mean and SD) are percentages of hydrolyzed starch from initial starch of the pooled bolus samples (*n* = 12).

Starch hydrolysis rates of penne and spaghetti boluses were slower (0.3 ± 0.1%/min and 0.4 ± 0.1%/min, respectively) compared with bread and couscous (0.7 ± 0.0%/min and 0.6 ± 0.1%/min, respectively) (**Figure 7**). After 30 min incubation the percentages of hydrolyzed starch of the original starch content were 12.5 ± 1.9% in spaghetti, 9.9 ± 1.8% in penne, 19.8 ± 0.7% in bread, and 19.6 ± 1.9 % in couscous.

### Properties of in vitro gastric-digested boluses

#### Disintegration of food matrix after in vitro gastric digestion

Clear differences in the cumulative particle size distribution were detected between the samples after in vitro gastric digestion (Figure 5). In vitro gastric-digested pasta boluses contained large particles: particles of size 10–100 mm^2^ occupied 90% of the total particle area whereas the share of particles with size <10 mm^2^ was low. The bread bolus consisted mostly of small (<1 mm^2^) particles. The in vitro gastric-digested couscous bolus contained a higher proportion of large particles than the bread bolus but a clearly higher proportion of small particles than the pasta boluses. There were no clear differences between the spaghetti and penne samples in the particle size distribution after in vitro gastric digestion. The amounts of dry matter lost as the smallest particles (<0.02 mm^2^) in the turbid liquid after particle separation was the highest in the in vitro gastric-digested bread bolus (20%).

#### Microstructure of in vitro gastric-digested boluses

After in vitro gastric digestion, large particles with darkly stainable starch granules embedded in a protein matrix were still detected in the pasta boluses (Figure 6). The effect of pepsin was observed as the disappearance of the outer layers of pasta digesta particles with the most hydrolyzed starch granules. The outer layer of in vitro gastric-digested pasta boluses contained darkly stained starch granules within the protein network. Darkly stainable, little modified starch granules were also detected in the couscous bolus (Figure 6). The protein network was partly intact in the inner parts of the particles. The swollen and deformed starch granules mostly disappeared. The protein matrix of the bread bolus was mostly degraded and the starch granules were extensively hydrolyzed (Figure 6).

## Discussion

The results of this study elucidate how pasta structure delays starch digestion causing lower postprandial glucose, insulin, and C-peptide responses in humans compared with other commonly consumed grain products like bread and couscous. This seems to be because, although these other grain-based dishes are made with the exact same raw material, they differ extensively in the technological processes applied to obtain the final food product. The selection of the same starting ingredient (semolina) represents a key point of the study, and the choice of using the same semolina and the same ingredients (simply water and salt) for producing all the samples was made to reduce potential confounding factors such as botanical/agronomic confounders (e.g., different cultivars or agronomic conditions), technological confounders (e.g., different milling process and particle size of the flour), or nutritional confounders (e.g., addition of fats to bread or different quantity of proteins in the wheat semolina), which have all been shown to alter postprandial glucose and insulin responses.

The lower postprandial glucose responses observed for the pastas compared with the bread and couscous respectively are well supported by earlier studies showing that pasta has a lower impact on postprandial glycemia compared with many other carbohydrate-rich foods (11). Previously, several studies have shown that pasta products usually have a GI that falls between the low and the medium ranges (10, 19), with respect to medium or high values for couscous and bread (19–21). Moreover, a lower postprandial insulin response for pasta compared with bread has been reported by Holt and colleagues (22). Granfeldt and colleagues (17) observed lower peak values for glucose, insulin, and C-peptide, and lower glycemic, insulin, and C-peptide indexes for pasta products when compared with bread baked from the same ingredients.

The glucose release rate from food during digestion is the main factor influencing postprandial glycemic and insulinemic responses of cereal products (23). The overall slow starch hydrolysis rate of pasta compared with couscous and bread was seen in this study as slower starch hydrolysis of pasta BSs. This observation is in line with the results of Hoebler and colleagues (24), who showed that about half of the bread starch and only 25% of pasta starch was transformed into smaller molecules during oral digestion.

The slower starch hydrolysis rate can be explained by bolus properties like lower saliva amount, larger size and smooth-rimmed shape of particles, and compact microstructure where starch granules were entrapped in a strong gluten network. Despite the longer chewing time, less saliva had incorporated into pasta boluses compared with bread or couscous boluses. The lower absorption of saliva can be explained by the high initial moisture content of pasta and small surface area of the particles. The amount of saliva determines the amount of salivary α-amylase in a food bolus and therefore it has been suggested to be an important factor for the overall breakdown of starch in starch-based foods (3). The observation that pasta remained as large and smooth-rimmed particles after mastication is in line with the work of Hoebler and colleagues who showed that spaghetti largely retained its physical structure in mastication (24). Such particles (large and smooth-rimmed in shape) have a low surface-to-weight ratio and are thus generally more resistant to enzymatic action compared with small and shredded particles with a larger surface area. In contrast to pasta boluses, bread and couscous boluses had a larger surface area: bread bolus particles were mostly very small, and the remaining larger particles were shredded in shape; and couscous particles were smooth-rimmed but small. To conclude, pasta particles had less area for enzymatic action compared with bread or couscous.

The microstructure of pasta boluses was like that of cooked pasta (6): the core of particles contained partially gelatinized starch granules inside a gluten network whereas the outer layer contained areas with swollen and deformed starch granules. Couscous had similar features, with areas of deformed starch granules in the outer layer of the granules and somewhat unchanged granules in the core. In contrast, starch granules in bread (25) and its bolus were thoroughly swollen and deformed. More extensively hydrolyzed starch granules (appearing swollen and deformed) are more prone to enzymatic hydrolysis and this can further explain the postprandial differences between pastas, bread, and couscous.

The differences in particle size distribution between pastas, bread, and couscous were even more pronounced after in vitro gastric digestion compared with after mastication: pasta boluses contained more large particles whereas bread and couscous had disintegrated to very small particles. The large differences in particle size could influence gastric emptying rates of these foods in vivo and therefore also influence the rate of starch digestion in the duodenum (26). The light microscopy images showed that after in vitro gastric digestion, the core of the pasta particles had remained partially unchanged with starch encapsulated in the protein network; only the outer layers with swollen starch granules had disappeared. Similarly, the outer layer of couscous particles with swollen starch granules had disappeared, but the core of the particle, with the protein network preserved to some extent, remained. Conversely, the protein network had largely disappeared from the in vitro gastric-digested bread bolus and the starch had been extensively hydrolyzed. These results indicate that pasta has a strong protein network that is slowly digested and that also protects starch granules from enzymatic action. Although couscous had a strong protein network (as seen by microscopy), due to its small granule size, it was more prone to enzymatic action, thus resulting in smaller particles compared with the pasta boluses after in vitro digestion.

Two different types of pasta (spaghetti and penne), which represent the 2 best-selling types, were analyzed for potential differences due to shapes and sizes. This comparison demonstrated a lower glycemic response of the long pasta (spaghetti) compared with the short pasta (penne), as previously found (27, 28). There were no clear differences in the amount of saliva, particle size distribution, or starch hydrolysis rates of spaghetti and penne boluses that could explain the difference seen in postprandial responses. However, the light microscopy images showed that in the spaghetti bolus, the starch granules in the core of the particle seemed to be very tightly packed, whereas in the penne bolus there was more protein and air spaces between the granules. This could indicate that different processing steps in spaghetti and penne manufacturing influence the molecular organization of the core of the pasta and that could influence starch digestibility. However, this would warrant further investigation because it is also possible that the air spaces were caused by shrinkage of the starch granules due to the dehydration step of sample preparation for embedding.

As shown in this and previous studies, pasta elicits lower postprandial glycemic and insulinemic responses compared with foods with similar ingredients but different structures. Postprandial glucose responses of foods are important because diets containing foods eliciting lower responses favorably affect the risk of several chronic diseases and their risk factors (29). A positive association between dietary GI and the increased risk of type 2 diabetes, coronary heart disease, and colorectal, breast, and bladder cancers, as well as between dietary glycemic load (GL) and the risk of coronary heart disease, type 2 diabetes, and stroke has been described (29–33). Mechanistically, the reduced postprandial insulin response reduces stress on the pancreatic β cells, thus preserving β cell functionality and reducing insulin resistance (34); it also prevents the blood glucose concentration from falling below the baseline fasting level. This is beneficial because hypoglycemia can have a variety of negative effects (35). Pasta consumption has been found to be inversely associated with BMI in the Moli-sani and INHES (Italian Nutrition & HEalth Survey) observational studies (36). Another observational study on type 2 diabetes patients demonstrated that the consumption of pasta, within the limits recommended for total carbohydrates intake, is not associated with worsening of glucose control, measures of adiposity, and major cardiovascular risk factors (37). In a recent intervention study, the presence of pasta in a hypocaloric Mediterranean diet induced significant reduction in body weight; moreover, beneficial effects were also observed for glycemic control and lipid metabolism (38). Furthermore, in a systematic review and meta-analysis of randomized controlled trials, pasta in the context of low-GI dietary patterns has been found to reduce body weight and BMI compared with higher-GI dietary patterns (39).

This study has several strengths and some limitations worth highlighting. Firstly, all the samples tested within the study were formulated starting from the same ingredient (semolina) to avoid any confounders derived from the use of a different matrix. No other ingredients known to potentially interfere with the postprandial glycemic response in vivo (e.g., oils and/or sauces) were added to the pasta products, couscous, and bread, allowing monitoring specifically of the effect of the technological process on glucose, insulin, and C-peptide responses. Another strength of the postprandial studies is that both trials were powered for 2 primary outcomes (glucose and insulin). Thirdly, the study investigated thoroughly the structural changes and starch digestibility of pasta and other semolina products, ranging from the mastication process, to bolus properties and properties of in vitro gastric-digested boluses, and finally observing the postprandial responses. This approach allows a comprehensive overview of the reasons behind postprandial glucose and insulin responses. One limitation of the postprandial studies could be the absence of data on other satiety-related hormones, which could support the description of the mechanism of action linked to the pasta consumption.

In conclusion, pasta consumption results in lower acute postprandial glycemic and insulinemic responses, when compared with other durum semolina products, making pasta a healthier source of available carbohydrates. This characteristic is attributed to the pasta structure, which is slowly disintegrated in mastication and gastric digestion, as shown in this study.

## Supplementary Material

nxab361_Supplemental_File

## Notes

This work was financially supported by Barilla G. e R. F.lli S.p.A., Parma (Italy).

Author disclosures: LC is currently a Mitacs-Elevate postdoctoral fellow jointly funded by the Government of Canada and the Canadian Sugar Institute. She was previously employed as a casual clinical coordinator at INQUIS Clinical Research, Ltd (formerly Glycemic Index Laboratories, Inc), a contract research organization. All other authors report no conflicts of interest.

Supplemental Tables 1 and 2 and Supplemental Figure 1 are available from the “Supplementary data” link in the online posting of the article and from the same link in the online table of contents at https://academic.oup.com/jn.

SV and MD'A contributed equally to the manuscript.

Abbreviations used: BS, bolus sample; DS, dry sample; EMG, electromyography; GI, glycemic index.

## References

[1] Singh J , Dartois A, Kaur L. Starch digestibility in food matrix: a review. Trends Food Sci Technol. 2010;21(4):168–80.

[2] Singh H , Ye A, Ferrua MJ. Aspects of food structures in the digestive tract. Curr Opin Food Sci. 2015;3:85–93.

[3] Bornhorst GM , Singh RP. Bolus formation and disintegration during digestion of food carbohydrates. Compr Rev Food Sci Food Saf. 2012;11(2):101–18.

[4] Autio K , Parkkonen T, Fabritius M. Observing structural differences in wheat and rye breads. Cereal Foods World. 1997;42(8):702–5.

[5] Hébrard A , Oulahna D, Galet L, Cuq B, Abecassis J, Fages J. Hydration properties of durum wheat semolina: influence of particle size and temperature. Powder Technol. 2003;130(1):211–18.

[6] Petitot M , Abecassis J, Micard V. Structuring of pasta components during processing: impact on starch and protein digestibility and allergenicity. Trends Food Sci Technol. 2009;20(11):521–32.

[7] Zou W , Sissons M, Gidley MJ, Gilbert RG, Warren FJ. Combined techniques for characterising pasta structure reveals how the gluten network slows enzymic digestion rate. Food Chem. 2015;188:559–68. 26041231 10.1016/j.foodchem.2015.05.032

[8] Cunin C , Handschin S, Walther P, Escher F. Structural changes of starch during cooking of durum wheat pasta. LWT Food Sci Technol. 1995;28(3):323–8.

[9] Englyst HN , Veenstra J, Hudson GJ. Measurement of rapidly available glucose (RAG) in plant foods: a potential in vitro predictor of the glycaemic response. Br J Nutr. 1996;75(3):327–37. 8785208 10.1079/bjn19960137

[10] Scazzina F , Dall'Asta M, Casiraghi MC, Sieri S, Del Rio D, Pellegrini N, Brighenti F. Glycemic index and glycemic load of commercial Italian foods. Nutr Metab Cardiovasc Dis. 2016;26(5):419–29. 27103122 10.1016/j.numecd.2016.02.013

[11] Huang M , Li J, Ha MA, Riccardi G, Liu S. A systematic review on the relations between pasta consumption and cardio-metabolic risk factors. Nutr Metab Cardiovasc Dis. 2017;27(11):939–48. 28954707 10.1016/j.numecd.2017.07.005

[12] Chiavaroli L , Di Pede G, Dall'Asta M, Cossu M, Francinelli V, Goldoni M, Scazzina F, Brighenti F. The importance of glycemic index on post-prandial glycaemia in the context of mixed meals: a randomized controlled trial on pasta and rice. Nutr Metab Cardiovasc Dis. 2020;31(2):615–25. 33229200 10.1016/j.numecd.2020.09.025

[13] Welch RW , Antoine JM, Berta JL, Bub A, de Vries J, Guarner F, Hasselwander O, Hendriks H, Jakel M, Koletzko BV et al. Guidelines for the design, conduct and reporting of human intervention studies to evaluate the health benefits of foods. Br J Nutr. 2011;106(Suppl 2):S3–15. 22129662 10.1017/S0007114511003606

[14] ISO . Food products—determination of the glycaemic index (GI) and recommendation for food classification. ISO 26642:2010. International Organization for Standardization; 2010.

[15] Brighenti F , Benini L, Del Rio D, Casiraghi C, Pellegrini N, Scazzina F, Jenkins DJ, Vantini I. Colonic fermentation of indigestible carbohydrates contributes to the second-meal effect. Am J Clin Nutr. 2006;83(4):817–22. 16600933 10.1093/ajcn/83.4.817

[16] Pentikäinen S , Sozer N, Närväinen J, Sipilä K, Alam SA, Heiniö R-L, Paananen J, Poutanen K, Kolehmainen M. Do rye product structure, product perceptions and oral processing modulate satiety?. Food Qual Preference. 2017;60:178–87.

[17] Granfeldt Y , Björck I, Drews A, Tovar J. An in vitro procedure based on chewing to predict metabolic response to starch in cereal and legume products. Eur J Clin Nutr. 1992;46(9):649–60. 1396482

[18] Pentikäinen S , Sozer N, Närväinen J, Ylätalo S, Teppola P, Jurvelin J, Holopainen-Mantila U, Törrönen R, Aura A-M, Poutanen K. Effects of wheat and rye bread structure on mastication process and bolus properties. Food Res Int. 2014;66:356–64.

[19] Foster-Powell K , Holt SHA, Brand-Miller JC. International table of glycemic index and glycemic load values: 2002. Am J Clin Nutr. 2002;76(1):5–56. 12081815 10.1093/ajcn/76.1.5

[20] Atkinson FS , Foster-Powell K, Brand-Miller JC. International tables of glycemic index and glycemic load values: 2008. Diabetes Care. 2008;31(12):2281–3. 18835944 10.2337/dc08-1239 PMC2584181

[21] Foster-Powell K , Miller JB. International tables of glycemic index. Am J Clin Nutr. 1995;62(4):871S–90S. 7572722 10.1093/ajcn/62.4.871S

[22] Holt SH , Miller JC, Petocz P. An insulin index of foods: the insulin demand generated by 1000-kJ portions of common foods. Am J Clin Nutr. 1997;66(5):1264–76. 9356547 10.1093/ajcn/66.5.1264

[23] Englyst KN , Vinoy S, Englyst HN, Lang V. Glycaemic index of cereal products explained by their content of rapidly and slowly available glucose. Br J Nutr. 2003;89(3):329–40. 12628028 10.1079/BJN2002786

[24] Hoebler C , Karinthi A, Devaux MF, Guillon F, Gallant DJ, Bouchet B, Melegari C, Barry JL. Physical and chemical transformations of cereal food during oral digestion in human subjects. Br J Nutr. 1998;80(5):429–36. 9924264 10.1017/s0007114598001494

[25] Juntunen KS , Laaksonen DE, Autio K, Niskanen LK, Holst JJ, Savolainen KE, Liukkonen KH, Poutanen KS, Mykkänen HM. Structural differences between rye and wheat breads but not total fiber content may explain the lower postprandial insulin response to rye bread. Am J Clin Nutr. 2003;78(5):957–64. 14594782 10.1093/ajcn/78.5.957

[26] Bornhorst GM , Singh RP. Gastric digestion in vivo and in vitro: how the structural aspects of food influence the digestion process. Annu Rev Food Sci. 2014;5(1):111–32. 10.1146/annurev-food-030713-092346 24387607

[27] Wolever TM , Jenkins DJ, Kalmusky J, Giordano C, Giudici S, Jenkins AL, Thompson LU, Wong GS, Josse RG. Glycemic response to pasta: effect of surface area, degree of cooking, and protein enrichment. Diabetes Care. 1986;9(4):401–4. 3743316 10.2337/diacare.9.4.401

[28] Granfeldt Y , Björck I, Hagander B. On the importance of processing conditions, product thickness and egg addition for the glycaemic and hormonal responses to pasta: a comparison with bread made from ‘pasta ingredients’. Eur J Clin Nutr. 1991;45(10):489–99. 1782920

[29] Jayedi A , Soltani S, Jenkins D, Sievenpiper J, Shab-Bidar S. Dietary glycemic index, glycemic load, and chronic disease: an umbrella review of meta-analyses of prospective cohort studies. Crit Rev Food Sci Nutr. 2020:1–10. 10.1080/10408398.2020.1854168 33261511

[30] Livesey G , Livesey H. Coronary heart disease and dietary carbohydrate, glycemic index, and glycemic load: dose-response meta-analyses of prospective cohort studies. Mayo Clin Proc Innov Qual Outcomes. 2019;3(1):52–69. 30899909 10.1016/j.mayocpiqo.2018.12.007 PMC6410335

[31] Jenkins DJA , Dehghan M, Mente A, Bangdiwala SI, Rangarajan S, Srichaikul K, Mohan V, Avezum A, Díaz R, Rosengren A et al. Glycemic index, glycemic load, and cardiovascular disease and mortality. N Engl J Med. 2021;384(14):1312–22. 33626252 10.1056/NEJMoa2007123

[32] Augustin LS , Kendall CW, Jenkins DJ, Willett WC, Astrup A, Barclay AW, Bjorck I, Brand-Miller JC, Brighenti F, Buyken AE et al. Glycemic index, glycemic load and glycemic response: an International Scientific Consensus Summit from the International Carbohydrate Quality Consortium (ICQC). Nutr Metab Cardiovasc Dis. 2015;25(9):795–815. 26160327 10.1016/j.numecd.2015.05.005

[33] Blaak EE , Antoine JM, Benton D, Bjorck I, Bozzetto L, Brouns F, Diamant M, Dye L, Hulshof T, Holst JJ et al. Impact of postprandial glycaemia on health and prevention of disease. Obes Rev. 2012;13(10):923–84. 22780564 10.1111/j.1467-789X.2012.01011.x PMC3494382

[34] Scazzina F , Dei Cas A, Del Rio D, Brighenti F, Bonadonna RC. The β-cell burden index of food: a proposal. Nutr Metab Cardiovasc Dis. 2016;26(10):872–8. 27381989 10.1016/j.numecd.2016.04.015

[35] Ludwig DS . The glycemic index: physiological mechanisms relating to obesity, diabetes, and cardiovascular disease. JAMA. 2002;287(18):2414–23. 11988062 10.1001/jama.287.18.2414

[36] Pounis G , Castelnuovo AD, Costanzo S, Persichillo M, Bonaccio M, Bonanni A, Cerletti C, Donati MB, de Gaetano G, Iacoviello L. Association of pasta consumption with body mass index and waist-to-hip ratio: results from Moli-sani and INHES studies. Nutr Diabetes. 2016;6(7):e218. 27376700 10.1038/nutd.2016.20 PMC4973136

[37] Vitale M , Masulli M, Rivellese AA, Bonora E, Babini AC, Sartore G, Corsi L, Buzzetti R, Citro G, Baldassarre MPA et al. Pasta consumption and connected dietary habits: associations with glucose control, adiposity measures, and cardiovascular risk factors in people with type 2 diabetes—TOSCA.IT Study. Nutrients. 2019;12(1):101. 10.3390/nu12010101 PMC7019547 31905885

[38] Rosi A , Tesan M, Cremonini A, Biasini B, Bicchieri L, Cossu M, Brighenti F, Dall'Aglio E, Scazzina F. Body weight of individuals with obesity decreases after a 6-month high pasta or low pasta Mediterranean diet weight-loss intervention. Nutr Metab Cardiovasc Dis. 2020;30(6):984–95. 32402585 10.1016/j.numecd.2020.02.013

[39] Chiavaroli L , Kendall CWC, Braunstein CR, Blanco Mejia S, Leiter LA, Jenkins DJA, Sievenpiper JL. Effect of pasta in the context of low-glycaemic index dietary patterns on body weight and markers of adiposity: a systematic review and meta-analysis of randomised controlled trials in adults. BMJ Open. 2018;8(3):e019438. 10.1136/bmjopen-2017-019438 PMC5884373 29615407

