# Supplementary material for: Pasta Structure Affects Mastication, Bolus Properties, and Postprandial Glucose and Insulin Metabolism in Healthy Adults
Source: J Nutr. 2021 Oct 20;152(4):994–1005. doi: 10.1093/jn/nxab361 (PMC8971003; doi:10.1093/jn/nxab361)

**Pasta structure affects mastication, bolus properties and postprandial glucose and insulin metabolism in healthy subjects.**

**Vanhatalo et al., Online Supplementary Material.**

**Supplementary Figures**

Supplementary Figure 1. The flow diagram of the enrolment for *in vivo* post-prandial study 1 (A) and *in vivo* post-prandial study 2 (B).

**A.**

**
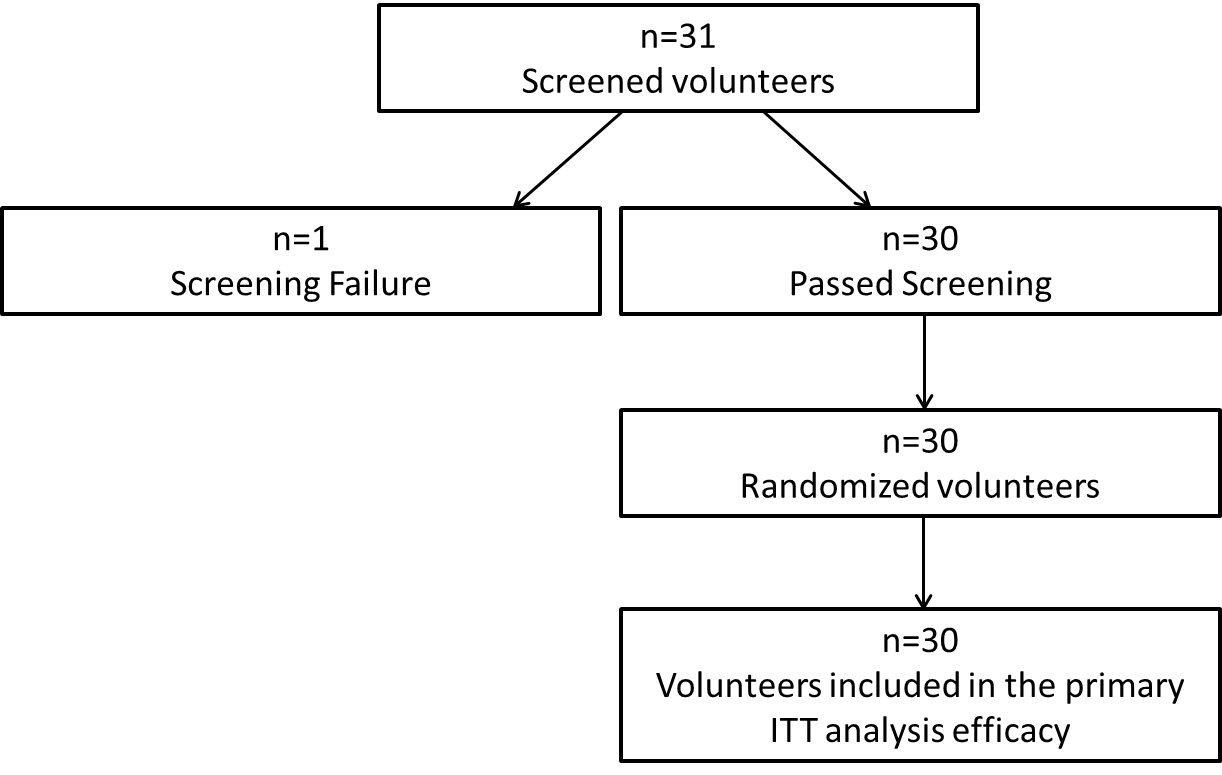
**

**B.**


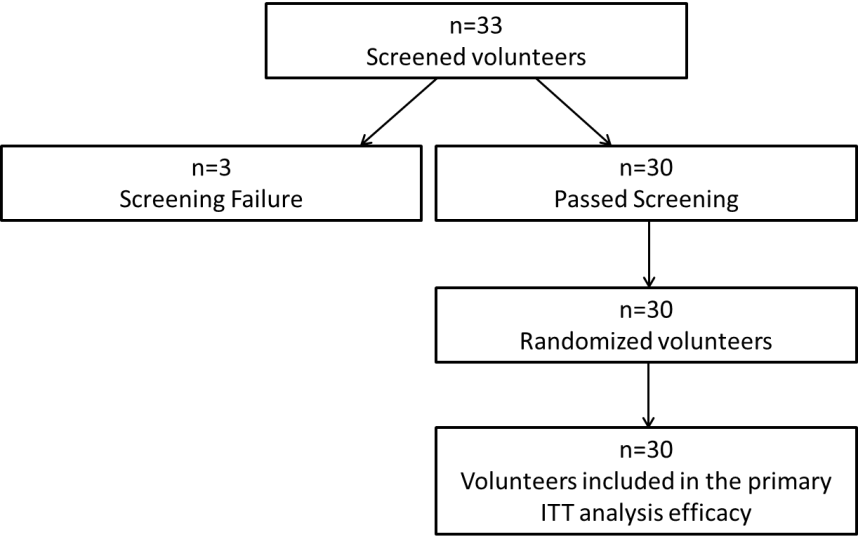

Supplement: nxab361_Supplemental_File [file nxab361_supplemental_file.zip › Online Supplementary Material_Figure_v2.docx]
