# Supplementary material for: Pasta Structure Affects Mastication, Bolus Properties, and Postprandial Glucose and Insulin Metabolism in Healthy Adults
Source: J Nutr. 2021 Oct 20;152(4):994–1005. doi: 10.1093/jn/nxab361 (PMC8971003; doi:10.1093/jn/nxab361)
Supplement: nxab361_Supplemental_File [file nxab361_supplemental_file.zip › Online Supplementary Material_Tables_v2.docx]

**Pasta structure affects mastication, bolus properties and postprandial glucose and insulin metabolism in healthy subjects.**

**Vanhatalo et al., Online Supplementary Material.**

**Supplementary Tables**

Supplemental Table 1. Characteristic of volunteers enrolled in post-prandial *in vivo* studies. Values are means ± SD.

|  | *in vivo* study 1 | *in vivo* study 2 |
| --- | --- | --- |
| *n* of subjects | 30 (16 F) | 30 (17 F) |
| Age (years) | 25 (17)^*^ | 29 (17)^*^ |
| Weight (kg) | 68.0 (23.7)^*^ | 66.6 ± 12.6 |
| BMI (kg/m^2^) | 23.9 ± 2.6 | 23.0 ± 3.0 |
| Waist circumference (cm) | 81.9 ± 9.8 | 77.9 ± 8.6 |
| Systolic pressure (mm Hg) | 111 ± 12 | 110 (16)^*^ |
| Diastolic pressure (mm Hg) | 72 ± 10 | 74 ± 8 |
| Blood glucose (mmol/L) | 4.3 ± 0.5 | 4.3 ± 0.4 |
| Plasma insulin (mU/L) | 10.6 (4.5)^*^ | 11.9 (4.3) |
| Plasma c-peptide (nmol/L) | 0.6 ± 0.2 | 0.5 (0.2)^*^ |

F=female; *Parameters not normally distributed and expressed as median (interquartile range).

Supplemental Table 2. Background information about the participants enrolled in mastication study. Values are means ± SD.

|  | All (n=26) | Males (n=11) | Females (n=15) |
| --- | --- | --- | --- |
| Age (years) | 36.7 ± 12.5 | 34.5 ± 13.7 | 38.3 ± 11.3 |
| BMI (kg/m^2^) | 23.7 ± 2.5 | 23.6 ± 3.3 | 23.8 ± 1.6 |
